# Myelin Oligodendrocyte Glycoprotein Antibody-Associated Disease (MOGAD) Following Adenovirus Meningitis After First Delivery: Case Report and Literature Review

**DOI:** 10.7759/cureus.48364

**Published:** 2023-11-06

**Authors:** Takashi Saito, Rieko Suzuki, Noboru Imai, Masahiro Serizawa

**Affiliations:** 1 Department of Neurology, Japanese Red Cross Shizuoka Hospital, Shizuoka, JPN

**Keywords:** mogad, neuromyelitis optica spectrum disorder (nmosd), covid-19 vaccines, covid−19, mutiple sclelosis, pregnancy, demyelinating diseases, autoimmune diseases of the nervous system, adenovirus, adem

## Abstract

Myelin oligodendrocyte glycoprotein antibody-associated disease (MOGAD) is a group of central nervous system (CNS) demyelinating diseases caused by autoantibodies against myelin oligosaccharide protein (MOG), a myelin sheath component protein, and present with a variety of symptoms, including optic neuritis, myelitis, acute disseminated encephalomyelitis (ADEM), brainstem encephalitis, and corticobasal encephalitis. It is currently unknown at what point in life MOGAD can develop or how it can be triggered by autoimmune mechanisms. Here, we report a case of a mature woman who suffered from adenoviral meningitis one month after childbirth and developed MOGAD but was able to return to child rearing with high-dose methylprednisolone therapy. This case suggests that the risk of developing MOGAD early after childbirth may be increased. The case also suggested that adenoviral infection may be involved in the development of MOGAD.

## Introduction

Myelin oligodendrocyte glycoprotein antibody-associated disease (MOGAD) is a general term for a group of demyelinating diseases of the central nervous system (CNS) that causes various symptoms, such as optic neuritis, myelitis, acute disseminated encephalomyelitis (ADEM), brainstem encephalitis, and cerebral cortical encephalitis due to autoantibodies against myelin oligodendrocyte glycoprotein (MOG), one of the myelin sheath component proteins [1,2]. However, MOG antibodies are now identified routinely in adults owing to the improvement of antibody testing technology. Therefore, it is not yet known at what point in life MOGAD can develop or how the autoimmune mechanism is triggered.

The impact of MOGAD on the onset and exacerbation of MOGAD [3,4], especially in the postpartum period in women, is not known, and although an association between viral infection and the onset of ADEM and subsequent MOGAD has been suggested, it is not known which specific viruses may induce the risk. Furthermore, there are still few clinical reports describing how symptoms, laboratory findings, and imaging findings change after patients diagnosed with MOGAD relatively early after the onset of symptoms receive immunotherapy.

Here, we report the clinical course of a case of a young woman who developed adenovirus meningitis as early as one month after her first childbirth, followed by MOGAD, and offer certain suggestions for the clinical qustion based on previous reports.

## Case presentation

A 30-year-old woman with no previous neurological history developed fever, headache, and urinary retention one month after the birth of her first child and visited her physician. She was admitted to the hospital to investigate the cause. Cerebrospinal fluid (CSF) examination revealed an elevated lymphocyte-dominant cell count, elevated protein concentration, and normal sugar concentration (Table 1).

**Table 1:** Trends in CSF examination results. The number of days since the onset of fever and headache is presented in the top row; IVMP was performed on a five-day schedule from day 35, and CSF test results began to improve. Mono: mononuclear cell; Poly: polynuclear cell; TP: total protein; Glu: glucose

Cerebrospinal fluid (CSF)	Day 5	Day 25	Day 53		Standard value
Cell counts	32	79	11	/mm^3^	0-5
Mono	72	95	88	%	
Poly	28	5	12	%	
TP	48	73	18	mg/dL	15-40
Glu	52	39	61	mg/dL	50-70

**Figure 1:**
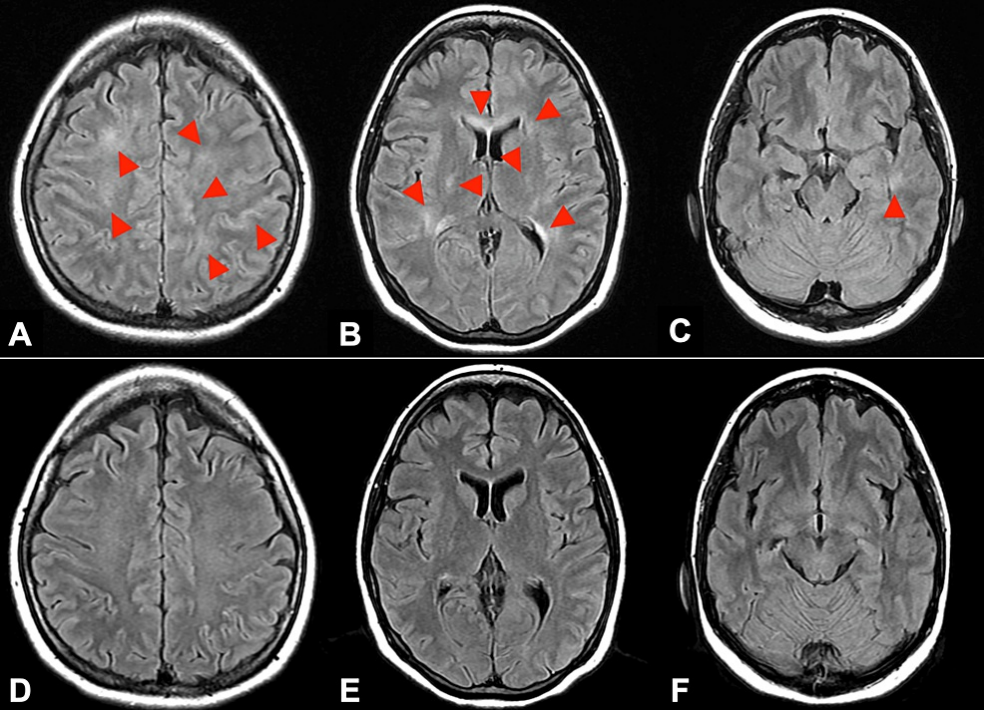
Brain MRI images of fluid-attenuated inversion recovery (FLAIR) before and after steroid pulse therapy. (A–C) were obtained before steroid pulse therapy. Brain MRI of the parietal level (A), basal ganglia level (B), and midbrain level (C) images showing hyperintensity of the subcortical white matter, deep white matter, and corpus callosum (red arrows). (D–F) were obtained after steroid pulse therapy. Each levels of brain MRI revealed almost complete reversal of previously observed high signals.

The patient was diagnosed with adenovirus meningitis after she tested positive for type C adenovirus with spinal fluid PCR test. After about 20 days of conservative treatment with external fluid infusion and rest, headache and fever worsened, with both hands and fingers becoming dull to the touch and showed decreased grip strength. The CSF examination revealed a further worsening (Table 1). The CSF IgG index was 0.72, and oligoclonal bands were negative. The patient was referred to our hospital due to worsening of clinical symptoms and laboratory findings. At the time of presentation, she was found to have symmetrical sensory deficits in the bilateral palms (decreased tactile, thermal, and painful sensations, no deep sensory abnormalities), as well as in the abdomen (Th10-12 region) and soles of the feet. Nerve conduction studies showed no obvious slowing of motor-sensory nerve conduction, prolonged distal latency, or decreased frequency of F waves. MRI of the brain revealed high signals in the subcortical white matter, deep white matter, and corpus callosum on fluid-attenuated inversion recovery (FLAIR) images (Figure 1), but no contrast effect.

**Figure 2:**
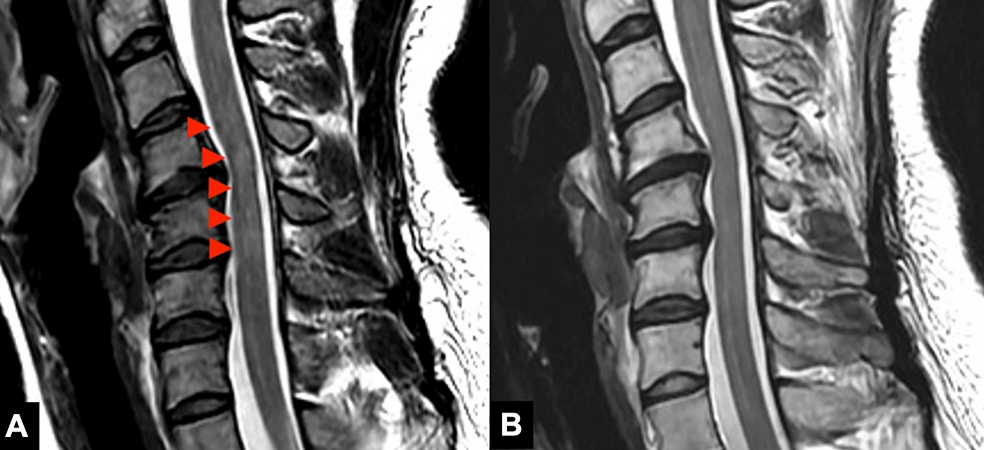
Cervical MRI T2-weighted images before and after steroid pulse therapy Pretreatment images showed T2-weighted high signal at the level of cervical vertebral bodies 4-5 (A), which resolved slowly three months after treatment (B).

Cervical MRI revealed a cervical herniation at C4/5 and a high-signal area on T2-weighted images at the same level of the spinal cord (Figure 2). She tested negative for serum HIV-1 and HIV-2 antigens and antibodies, anti-HTLV-1 antibody, VZV-IgM and IgG, *Mycobacterium tuberculosis*-specific interferon gamma test, anti-nuclear antibody, anti-citrullinated peptide antibody, anti-neutrophil cytosolic antibody, anti-glutamic acid decarboxylase antibody, anti-cardiolipin antibody, anti-ganglioside antibodies (including IgM and IgG for GM1, GM2, GM3, GD1a, GD1b, GD3, GT1b, GQ1b, and Gal-C, respectively), and anti-aquaporin 4 antibody. However, anti-MOG antibody was positive with a titer of 1:256. Ophthalmologic examination revealed a central flicker value of 42/43 Hz, bilateral 1.2 corrected visual acuity, and no evidence of optic neuritis. Based on these findings, MOGAD was diagnosed according to the diagnostic criteria proposed by the 2023 International MOGAD Panel [5].

Symptoms slowly worsened, and the extent of sensory disturbance expanded from the sole to the ascending part of the foot. However, it began to improve including severity of fever and headache after intravenous pulse methylprednisolone (IVMP) was started, and urinary retention disappeared. Post-steroidal therapy with prednisolone (1 mg/kg), which was tapered over time, ameliorated sensory disturbances. CSF examination results improved (Table 1). MRI of the brain revealed an almost complete reversal of previously observed high signals in the subcortical white matter, deep white matter, and corpus callosum on FLAIR images (Figure 1). The Mini-Mental State Examination (MMSE) improved from an initial score of 28 to 30 after two weeks of treatment. The patient was discharged on the 44th day of hospitalization and returned to childcare.

Three months after treatment, the titer of the anti-MOG antibody decreased to 1:16, and the high signal previously seen on the T2-weighted images on cervical MRI resolved (Figure 2).

## Discussion

The impact of MOGAD on the onset and exacerbation of MOGAD [3,4], especially in the postpartum period in women, is not known, and although an association between viral infection and the onset of ADEM and subsequent MOGAD has been suggested, it is not known which specific viruses may induce the risk. Furthermore, there are still few clinical reports describing how symptoms, laboratory findings, and imaging findings change after patients diagnosed with MOGAD relatively early after the onset of symptoms receive immunotherapy. Therefore, we report the clinical course of a case of a young woman who developed adenovirus meningitis as early as one month after her first childbirth, followed by MOGAD. 

Limited studies have reported a significantly decreased risk of recurrence during and immediately after pregnancy among women of childbearing age diagnosed with MOGAD [3]. On the other hand, it has been suggested that the risk of rebound increases postpartum [4], and sufficient evidence remains to be established due to the small number of cases. The present case is a new onset after first delivery, and it is not known whether the association is direct or indirect, but if these immunological mechanisms are clarified, the present case may logically be explained by a causal relationship.

Although there are no established diagnostic criteria for ADEM in adult patients, the clinical symptoms and imaging findings were consistent with ADEM itself. ADEM is one of the representative phenotypes of MOGAD in children [6]. Adenoviruses can cause ADEM and may also have fatal clinical outcomes in adults [7,8]. Reports suggested the development of MOGAD [9] and ADEM after adenovirus vector-based SARS-CoV-2 vaccination [10]. In all of these reports, MOGAD developed within a few months of the triggering event, similar to the clinical course of this case. Furthermore, it has been suggested that vector-based AstraZeneca (ChAdOx1S) vaccination is more likely to cause autoimmune CNS diseases than Pfizer (BNT162b2) vaccination [11,12]. ChAdOx1S is a platelet factor 4 (PF4) vaccine associated with other antibody-mediated diseases, such as vaccine-induced thrombosis and thrombocytopenia (VITT), caused by antibodies against platelet factor 4 (PF4). Furthermore, large clinical studies also confirmed a significant increase in the risk of antibody-mediated peripheral nerve diseases, such as Guillain-Barré syndrome and Bell's palsy, three weeks after ChAdOx1S vaccination [13,14]. The ChAdOx1S is a spike protein in the replication-deficient chimpanzee adenovirus vector. It has been hypothesized that the attachment of the adenoviral vector to heparan sulfate induces the binding of PF4 to heparan sulfate to neutralize it, resulting in a conformational change of the PF4 molecule that increased immunogenicity due to higher autoimmune reactivity [15]. In a systematic review, adenoviral vector vaccines were associated with the majority of ADEM and Neuromyelitis optica spectrum disorder /MOGAD cases, while mRNA vaccines were associated with a higher frequency of new onset and relapse of multiple sclerosis [16]. The accumulation of these reports suggests that adenovirus infection is selectively associated with the development of MOGAD, and this case was considered to be typical of that clinical course.

In addition, a retrospective study of patients who presented with optic neuritis symptoms showed a correlation between the time to initiate IVMP and the degree of visual recovery, suggesting the importance of early treatment [17]. Some experts have also reported that slow tapering of oral glucocorticoids over several months may reduce the risk of early recurrence [18]. In the present case, IVMP was initiated within a few weeks of onset of symptoms, and the patient responded well to treatment and quickly improved and returned to childcare. The treatment course was good, with no symptom flare-ups until three months after steroid administration, the titer of anti-MOG antibodies decreased to 1:16, and imaging studies showed that MRI high signal lesions in the brain and spinal cord remained resolved. However, careful follow-up is needed to prevent recurrence while slowly decreasing the dosage. The duration of immunotherapy for the prevention of attacks and relapses in patients with MOGAD is not clearly defined, and further studies are needed.

## Conclusions

This case report is aimed at a medical review of a young woman who developed adenovirus meningitis one month after her first delivery and was subsequently diagnosed with MOGAD. In this context, MOGAD may be associated with an increased risk of developing adenoviral meningitis early after childbirth. Adenovirus infection may be involved in the development of MOGAD. Nevertheless, early diagnosis and therapeutic intervention after the onset of MOGAD contributes to symptom improvement. Thus, testing with MOGAD in mind is considered for patients who present with such a variety of neurologic symptoms immediately after delivery.
